# Identification of novel clusters of co-expressing cytokines in a diagnostic cytokine multiplex test

**DOI:** 10.3389/fimmu.2023.1223817

**Published:** 2023-07-31

**Authors:** Daniel J. Polley, Penny Latham, May Y. Choi, Katherine A. Buhler, Marvin J. Fritzler, Mark L. Fritzler

**Affiliations:** ^1^ Eve Technologies Corporation, Calgary, AB, Canada; ^2^ Department of Medicine, Cumming School of Medicine, University of Calgary, Calgary, AB, Canada

**Keywords:** cytokines, clustering, multiplex assay, diagnostic test, immune activation, cytokine storm, inflammation

## Abstract

**Introduction:**

Cytokines are mediators of the immune system that are essential for the maintenance, development and resolution of immune responses. Beneficial immune responses depend on complex, interdependent networks of signaling and regulatory events in which individual cytokines influence the production and release of others. Since disruptions in these signaling networks are associated with a wide spectrum of diseases, cytokines have gained considerable interest as diagnostic, prognostic and precision therapy-relevant biomarkers. However, currently individual cytokines testing has limited value because the wider immune response context is often overlooked. The aim of this study was to identify specific cytokine signaling patterns associated with different diseases.

**Methods:**

Unbiased clustering analyses were performed on a clinical cytokine multiplex test using a cohort of human plasma specimens drawn from individuals with known or suspected diseases for which cytokine profiling was considered clinically indicated by the attending physician.

**Results and discussion:**

Seven clusters of co-expressing cytokines were identified, representing common patterns of immune activation. Common expression profiles of the cytokine clusters and preliminary associations of these profiles with specific diseases or disease categories were also identified. These findings increase our understanding of the immune environments underlying the clinical presentations of patients of inflammatory, autoimmune and neoplastic diseases, which could then improve diagnoses and the identification of evidence-based treatment targets.

## 1 Introduction

Cytokines are protein mediators of the immune system that have major impacts on the maintenance, activation, progression, regulation and resolution of immune responses (1). Cytokines can be broadly classified as pro-inflammatory or anti-inflammatory factors, as chemokines which regulate cell migration, or as stimulating factors that drive the proliferation, survival and differentiation of hematopoietic cells and their progenitors. These broad classifications are complicated by evidence that most cytokines are highly pleiotropic and can regulate diverse biological processes. The function of cytokines is dependent on the tissue, immune or inflammatory context and microenvironment, as factors that are pro-inflammatory in one context can be anti-inflammatory in another. For example, interleukin (IL)-4 is a major driver of inflammation in allergic diseases such as asthma (2), but has anti-inflammatory effects in autoinflammatory conditions such as rheumatoid arthritis and psoriasis (3). Additionally, cytokines can be characterized based on the type or pathways of immune response that they contribute to, although as noted above, these characterizations are variable based on their highly pleiotropic nature.

Broadly considered, immune activation can be classified as innate or adaptive. Cytokines that are induced, activated, and/or released following the recognition of molecular patterns associated with pathogens (pathogen-associated molecular patterns or PAMPs) or to tissue damage (damage-associated molecular patterns or DAMPs), such as the IL-1 family (IL-1α, IL-1β, IL-18, IL-33), IL-6, tumor necrosis factor α (TNFα), and interferons (IFN), contribute to innate immunity (4). Adaptive immunity refers to inflammation driven by lymphocytes (T and B cells). Different programs of adaptive immunity have evolved to confront different types of pathogens: type 1 immunity (driven by T helper (Th) 1 cells) targets intracellular pathogens such as viruses; type 2 immunity (Th2-mediated) targets multicellular pathogens such as helminths; and type 3 immunity (Th17-mediated) targets small extracellular pathogens. The T cell responses then orchestrate the release of inflammatory mediators and the recruitment of appropriate leukocytes to the site of inflammation. Dysregulation of type 1 and type 3 immune responses tends to result in autoimmune and autoinflammatory conditions, while dysregulation of type 2 immune responses tends to result in conditions associated with allergy, such as asthma (5). The dysregulation of these immune pathways often involves an unusual expression or release of cytokines. Individual cytokines have therefore been identified as diagnostic and therapeutic targets in a number of conditions, such as autoimmune and autoinflammatory diseases (6, 7), chronic inflammatory conditions (8), cancers (9), among others. As such, there is considerable interest in using circulating cytokine levels as complimentary diagnostic, prognostic or actionable biomarkers in inflammatory and autoimmune diseases (10).

An established methodology in the quantification of cytokines is the addressable laser bead (i.e., Luminex™) technology, which consists of highly sensitive bead-based multiplex assays that allow the simultaneous detection of up to 100 analytes using a low volume of biological fluid samples (1). In our laboratory (Eve Technologies Corporation, Calgary, AB), this technology platform is used to provide multiplex diagnostic tests quantifying levels of up to 71 circulating cytokines, chemokines and growth factors. However, the interpretation of a test result consisting of a large panel of analytes can be challenging, particularly when the biomarkers can have diverse and opposing biological effects in different immune contexts, and the immune responses that they regulate involve complex cascades of signaling events.

Our goal was to enhance the interpretation of our testing by reflecting the interaction of multiple analytes in the immune environment in our clinical results, which we aim to achieve by identifying patterns of commonly co-expressing cytokines by clustering analysis. Clustering analysis is an unbiased data mining technique that separates a dataset into groups based on the similarity of the individual datapoints. This type of analysis has become a common approach to identify cytokine signatures as diagnostic or prognostic markers in diseases such as COVID-19 (11), systemic lupus erythematosus (12), chronic lymphocytic leukemia (13), small cell lung cancer (14), among many others. By performing clustering analyses in a cohort of clinical specimens submitted for cytokine testing, our aim was to identify patterns of cytokine expression reflecting common signatures of immune activation in a diverse array of inflammatory, autoimmune, and neoplastic conditions which could then be used to aid clinicians in making diagnoses and in identifying potential treatment targets.

## 2 Materials and methods

### 2.1 Specimen collection

Plasma-EDTA specimens were collected for cytokine, chemokine and growth factor testing as requested by 88 health care providers and referral labs in eight Canadian provinces and territories and the USA, and submitted either to our laboratory (Eve Technologies, Calgary, AB) or to an affiliated laboratory (MitogenDx, Calgary, AB) between March 2021 and October 2022. All patient identities were anonymized and only demographic information (age, sex, reason for testing/diagnosis, location) was extracted from the test requisition forms. Information on disease severity or clinical outcomes associated with the specimens was not recorded. Specimens were received on dry ice and stored at -20°C until they were processed (typically within 3-5 days). Specimens that were provided with incomplete patient demographic information (age, sex, location), that did not meet pre-analytic quality criteria (e.g., grossly hemolyzed or lipemic) and/or that demonstrated possible test interferences (e.g., human anti-mouse antibody (HAMA)-induced false positive signals (15)) were not included in the analysis.

### 2.2 Biomarker measurements

Analysis of patient specimens for clinical diagnostics was performed using commercially available MilliporeSigma MILLIPLEX^®^ base kits [designated Research Use Only (RUO)] as described below, on Luminex 100/200™ instruments (Luminex/DiaSorin, Saluggia, Italy), with Bio-Plex Manager™ (BPM) software (BioRad, Hercules, CA). Eve Technologies’ Cytokine, Chemokine, Growth Factor 71-Plex Clinical RUO Test (HD71-CLIN) simultaneously detect plasma levels of 71 analytes on two separate panels as per the manufacturer’s instructions for use: 1.) the Human Cytokine 48-Plex Discovery Assay^®^ (HD48; Millipore MILLIPLEX^®^ Human Cytokine/Chemokine/Growth Factor Panel AImmunology Multiplex Assay, Cat. #HCYTA-60K), and 2.) the Human Cytokine 23-Plex Discovery Assay^®^ (HD23; Millipore MILLIPLEX^®^ Human Cytokine/Chemokine Magnetic Bead Panel IIImmunology Multiplex Assay, Cat. #HCYP2MAB-62K). Each specimen on each panel was run in singlet on the same analyzers by Eve Technologies, which is certified to perform high complexity laboratory testing under the Clinical Laboratory Improvement Amendments (CLIA).

### 2.3 Statistical analysis

Since a relative absence (or low levels) of any particular analyte could be meaningful with respect to patterns of cytokine expression, datapoints with fluorescence values below the range of the standard curves (for which concentration values are unable to be interpolated) were assigned a value of 0.01 pg/ml and were included in the analysis. The concentration values derived from the standard curves for each analyte were transformed into percentile ranks to bring the entire dataset within a similar range for the clustering analyses and heatmap generation.

### 2.4 Cytokine clustering analysis

K-means clustering was performed using R (version 4.2.2) with the base ‘kmeans’ function, and the resulting clusters were visualized with 2-dimensional cluster plots using the factoextra R package. The optimal number of clusters was estimated with both silhouette analysis and the elbow method (within-cluster sum of squared errors) using the factoextra package. Hierarchical clustering using Spearman rank correlation coefficients as the distance metric was performed using the base ‘hclust’ R function, and the resulting dendrogram was visualized using the factoextra package. Cytokine clusters were identified by partitioning the dendrogram according to the height metric provided by the factoextra package. Additionally, to assess correlations between every analyte, a correlation matrix plot of Spearman rank coefficients (calculated with the hmisc R package) was generated using the corrplot R package with only significant (p < 0.01) correlations plotted.

The approach of comparing the results of two separate clustering analyses, K-means and hierarchical clustering, was taken to identify more strongly clustering analytes, since the algorithms associated with different clustering methods can yield variable results (16). As each strategy has its own advantages and disadvantages (17), a consensus between the two different clustering methods can be taken as an indication of the validity of the clusters, which can be corroborated with an assessment of the potential physiological significance of each cluster.

### 2.5 Patient clustering analysis

To assess the potential clinical or pathophysiological significance of the cytokine clusters, as well as associations between patterns of cytokine signaling and demographic characteristics (age and sex), the patient data were clustered and visualized as a heatmap using the ComplexHeatMap R package (18). The analytes were grouped into the consensus clusters, the patients were grouped by age, since circulating cytokine levels have been shown to be influenced by age (19), and the different patient age groups were clustered separately with hierarchical clustering using Spearman rank correlation coefficients as the distance metric. Groups of patients with similar cytokine profiles were identified by partitioning the dendrograms based on visual inspection of the heatmaps and dendrograms. To characterize the cytokine cluster expression profiles for each of the patient clusters, the median percentile rank values of all of the analytes within each cytokine cluster were calculated. To illustrate the relative expression levels of the cytokines within each cluster, the percentile distributions were partitioned into quintiles, with median percentile rank values of 0-20% designated to be ‘low’; 20-40% ‘moderate low’; 40-60% ‘median’; 60-80% ‘moderate high’, and 80-100% ‘high’.

To assess differences in demographic information of each patient cluster compared to their respective total age group cohorts, the following tests were performed: The Kruskal-Wallis test, with Dunn’s test to correct for multiple comparisons, was used to compare the median age values using Graphpad Prism version 9.5.0; the binomial test was used to compare the sex ratios using the base ‘binom.test’ R function.

Additionally, to examine the association of patterns of cytokine expression with specific conditions, a heatmap was generated using only the patients that had diagnoses or reasons for testing available, and limited to conditions associated with at least two patients. Patients associated with the same condition, or with a similar category of conditions (e.g., lymphoproliferative diseases, or eosinophilic conditions, etc.), were manually grouped together, patients within each group were clustered using hierarchical clustering, and all clustered groups were combined into a single heatmap for comparison.

## 3 Results

### 3.1 Patient demographics

254 specimens drawn from unique individuals were included in the analysis, including 66 children and 188 adults. The median age of the total cohort was 39 years, with a range from 6 months to 87 years old. The pediatric cohort had a median age of 8.5 years with a range from 6 months to 17 years old, and the adult cohort had a median age of 46 years with a range of 18-87 years old. The sex ratio of the individuals was approximately equal with 128 males and 126 females, with 36 males and 30 females in the pediatric cohort, and with 92 males and 96 females in the adult cohort. Patient demographic information in this cohort is summarized in **Table 1**.

**Table 1:** Demographics of Study Cohort.

Characteristic	N=
**Sample size**	254
Age (years)	
**Median (range)** 39 (0.5 - 87)	
0-17	66
18-39	63
40-59	80
60+	45
Sex	
Male	128
Female	126
0-17 years	
Male	36
Female	30
18-39 years	
Male	26
Female	37
40-59 years	
Male	42
Female	38
60+ years	
Male	24
Female	21
Location	
AB	69
BC	59
Other	12
QC	101
USA	13

### 3.2 Cytokine clustering

**Figure 1:**
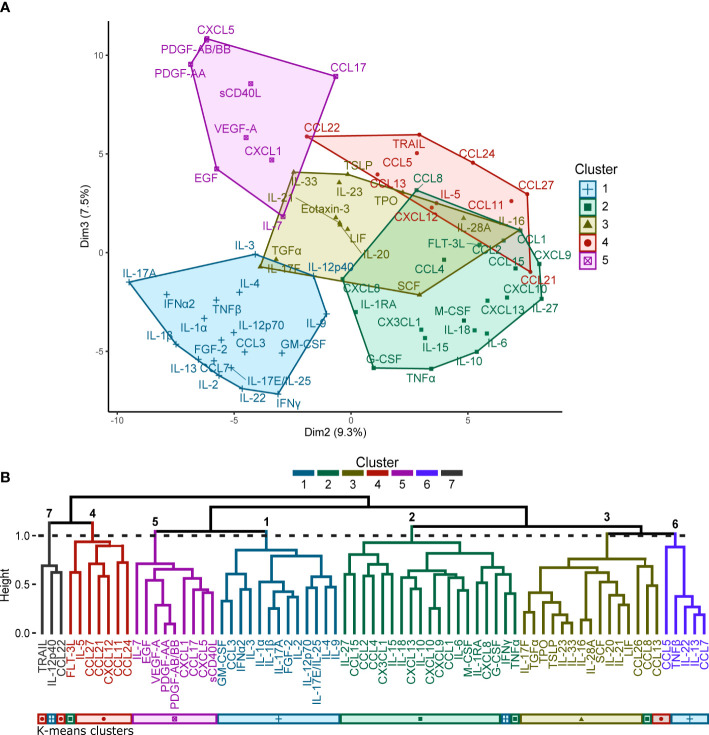
**(A)** K-means clustering performed for the 71 cytokines represented as a 2-dimensional cluster plot based on a principal component analysis. Silhouette and elbow method analyses determined that the optimal number of clusters for K-means clustering was five. **(B)** Hierarchical clustering performed for the 71 cytokines represented by a dendrogram. Seven clusters were identified by partitioning the dendrogram at the height of the dashed line. A comparison to the clusters generated by the K-means analysis is provided by the coloured bars beneath the dendrogram.

Silhouette and elbow method analyses of the percentile rank data yielded an estimated optimal number of five clusters for this dataset for K-means clustering (**Figure 1A**). Seven clusters were identified in the hierarchical clustering analysis by partitioning the dendrogram (**Figure 1B**). Comparisons of the clusters generated by each of the clustering methods yielded seven consensus groupings of consistently co-clustering cytokines, with twelve analytes (CCL5, CCL8, CCL7, CCL13, CCL22, FLT-3L, IFNγ, IL-12p40, IL-13, IL-22, TNFβ, TRAIL) that were placed in separate clusters by the different clustering methods (summarized in **Table 2**). Clusters 1-3 and 6 contain mostly pro-inflammatory factors and hematopoietic growth factors; cluster 4 contains mostly chemokines; cluster 5 contains several major growth factors; the functional link between the analytes in cluster 7 is unclear.

**Table 2:** Consensus clusters of the two cytokine clustering analyses.

Cluster 1	Cluster 2	Cluster 3	Cluster 4	Cluster 5	Cluster 6	Cluster 7
CCL3	CCL1	CCL26	CCL11	CXCL1	*CCL7 (H)*	*CCL22 (H)*
FGF-2	CCL2	IL-16	CCL15	CXCL5	*IL-13 (H)*	*IL-12p40 (H)*
GM-CSF	CCL4	IL-17F	CCL21	CXCL17	*IL-22 (H)*	*TRAIL (H)*
IFNα2	CX3CL1	IL-20	CCL24	EGF	*TNFβ (H)*	
IL-1α	CXCL8	IL-21	CCL27	IL-7	*CCL5 (H)*	
IL-1β	CXCL9	IL-23	CXCL12	PDGF-AA		
IL-2	CXCL10	IL-28A	IL-5	PDGF-AB/BB		
IL-3	CXCL13	IL-33	*CCL5 (K)*	sCD40L		
IL-4	G-CSF	LIF	*CCL13 (K)*	VEGF-A		
IL-9	IL-1RA	SCF	*CCL22 (K)*			
IL-12p70	IL-6	TGFα	*FLT-3L (H)*			
IL-17A	IL-10	TPO	*TRAIL (K)*			
IL-17E/IL-25	IL-15	TSLP				
*TNFβ (K)*	IL-18	*CCL8 (H)*				
*CCL7 (K)*	IL-27	*CCL13 (H)*				
*IFNγ (K)*	M-CSF					
*IL-12p40 (K)*	TNFα					
*IL-13 (K)*	*CCL8 (K)*					
*IL-22 (K)*	*FLT-3L (K)*					
	*IFNγ (H)*					

Cytokines/growth factors: EGF, epidermal growth factor; FGF, fibroblast growth factor; FLT-3L, Fms-related tyrosine kinase 3 ligand; IFN, interferon; IL, interleukin; G-CSF, granulocyte colony stimulating factor; GM-CSF, granulocyte macrophage colony stimulating factor; LIF, leukemia inhibitory factor; M-CSF, macrophage colony stimulating factor; PDGF, platelet-derived growth factor; SCF, stem cell factor; TGF, transforming growth factor; TPO, thrombopoietin; TNF, tumor necrosis factor; TRAIL, TNF-related apoptosis-inducing ligand; TSLP, thymic stromal lymphopoietin; VEGF, vascular endothelial growth factor.

Analytes that were placed into different clusters by the different clustering methods are listed in both clusters and indicated with italic text, and the clustering method associated with the specific cluster is indicated in parentheses (H-hierarchical, K-K-means).

**Figure 2:**
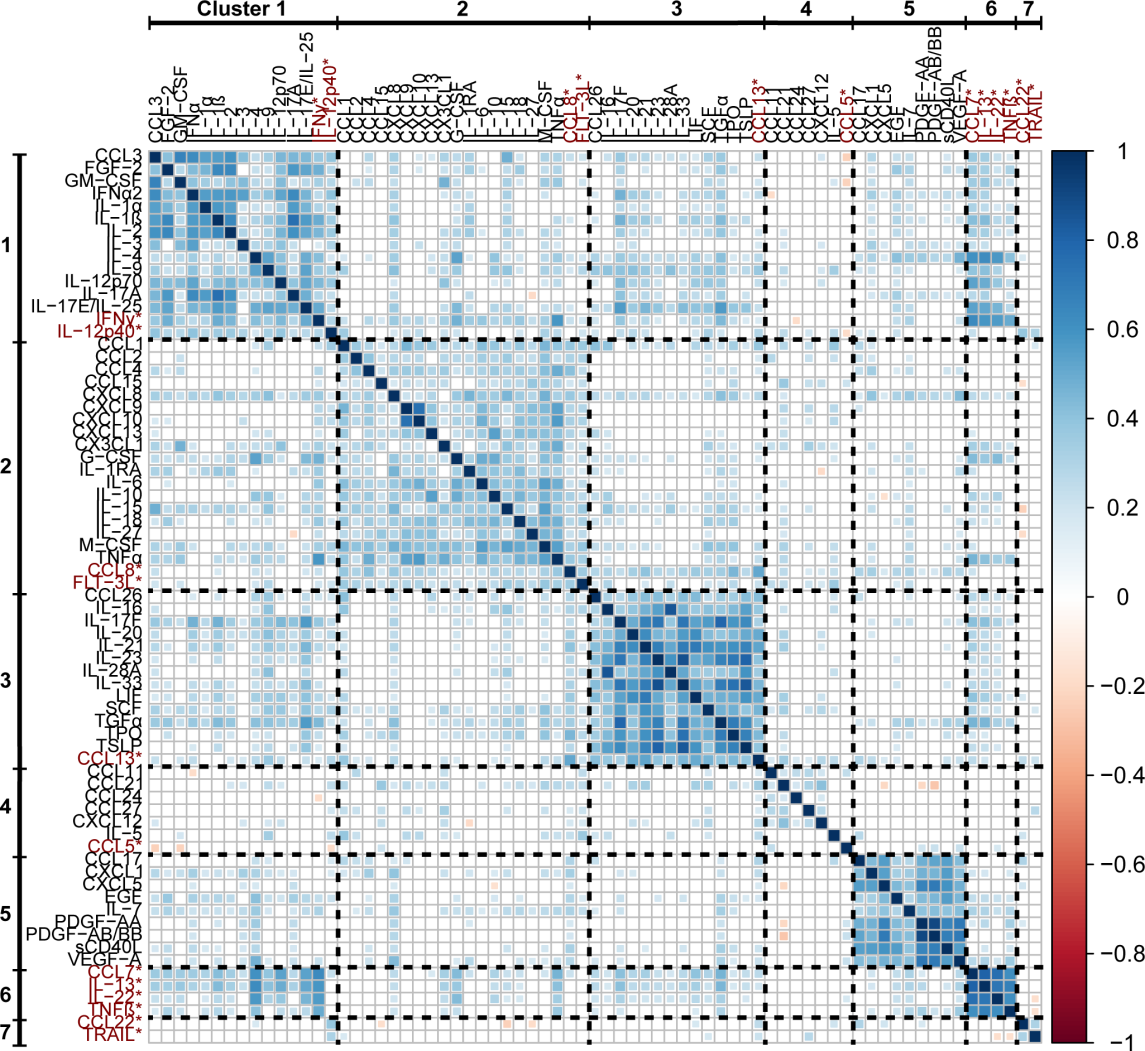
A correlation matrix of Spearman rank coefficients between the percentile rank data of each analyte. Only significant correlations (p < 0.01) are plotted, non-significant correlations are left blank. Analytes that were placed into separate clusters by the different clustering methods are included in the clusters in which they had the highest associated Spearman rank coefficients, and are highlighted with red text and asterisks (*).

Significant correlations (p < 0.01) between each analyte are visualized in a correlation plot (**Figure 2**) where positive correlations are coloured blue, negative correlations are coloured red, and non-significant correlations are left white in the plot. The analytes that were grouped into different clusters by the two clustering methods were placed in the cluster associated with the highest Spearman coefficients. Each cytokine cluster (separated in the plot by dashed lines) demonstrates strong intra-cluster correlations, visualized as blue squares along the diagonal axis of the plot.

### 3.3 Patient clustering

**Figure 3:**
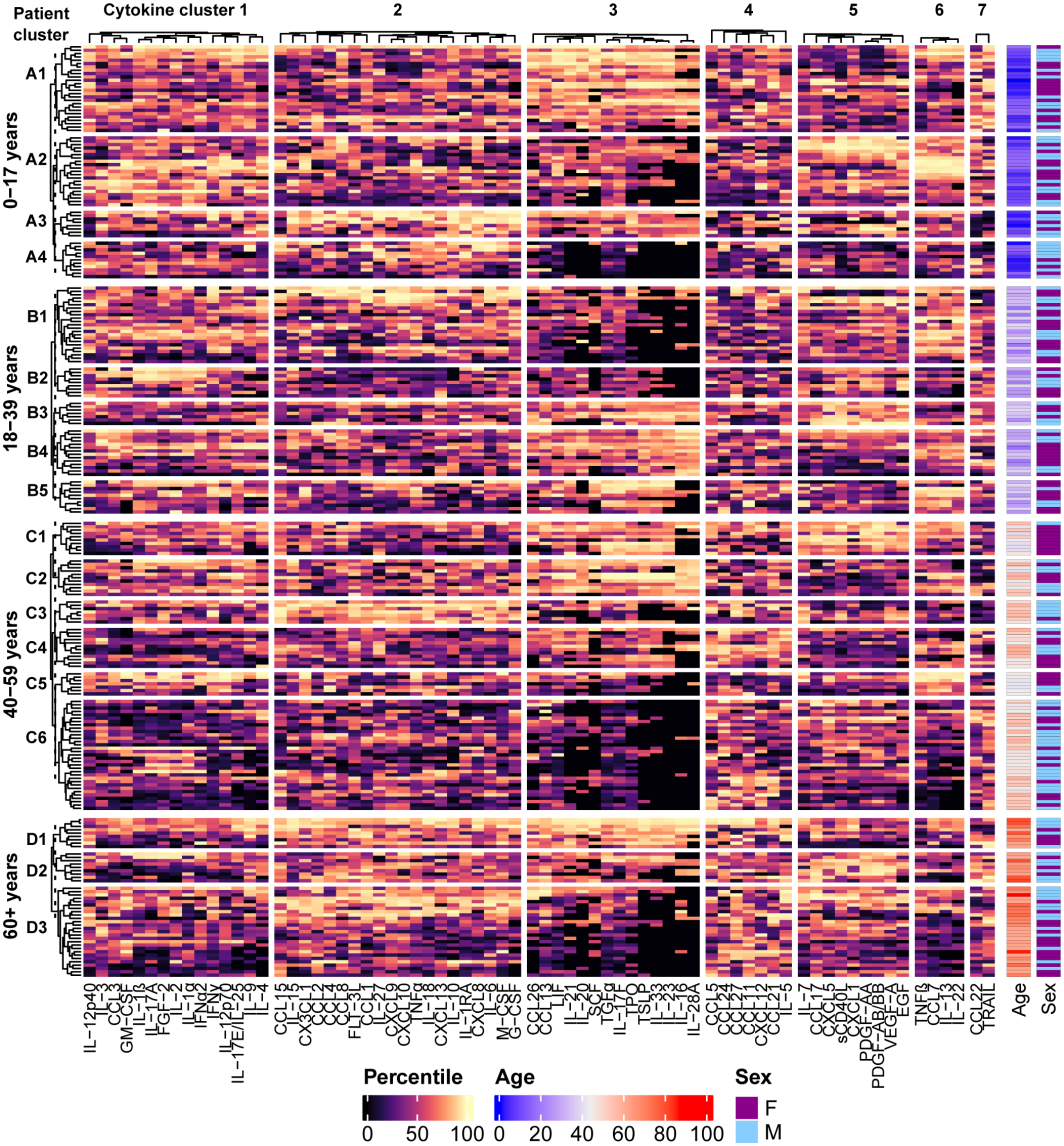
Distinct patterns of cytokine signaling are revealed by performing hierarchical clustering of the patients using the percentile rank data for each analyte. Individuals were grouped by age and the different age groups were clustered separately. Four clusters in the 0-17 year age group, five clusters in the 18-39 year age group, six clusters in the 40-59 year age group, and 3 clusters in the 60+ year age group were identified by partitioning the dendrograms at the height of the dashed line, and cytokine signatures are identified by their associated patterns in the heatmap. Demographic information for each patient is presented in the annotations on the right side of the heatmap.

Patients were grouped based on age and the different age groups were clustered separately, yielding four clusters for the pediatric (0-17 years) age group (clusters A1-A4), five clusters for the 18-39 year age group (clusters B1-B5), six clusters for the 40-59 year age group (clusters C1-C6), and three clusters for the 60+ year age group (clusters D1-D3), each consisting of patient specimens with similar cytokine expression patterns (**Figure 3**). The cytokine profiles of each patient cluster, characterized by the median percentile rank values of each cytokine cluster, along with the known diagnoses/reasons for testing associated with the individuals in each patient cluster, are summarized in **Table 3** (pediatric) and **Table 4** (adult). Similar conditions were grouped into the same clusters in some instances, for example there were multiple diagnoses of arthritis and fever in cluster A1, hemophagocytic lymphohistiocytosis (HLH) in clusters B1 and C3, adult-onset Still’s disease (AOSD) in clusters C3 and D3, and autoimmune/autoinflammatory conditions in cluster C1, etc. However, there were also several instances in which patients with the same condition were grouped into different clusters within each age group. For example, there were patients with AOSD that were grouped into clusters B1 and B5, C1 and C3, D2 and D3, etc. This finding could indicate the presence of multiple immune profiles associated with the same condition.

**Table 3:** Pediatric clusters.

Patient cluster	Diagnosis/Reason for testing	Cytokine cluster (median percentile rank)						
		1	2	3	4	5	6	7
0-17 years								
A1	Systemic arthritis/macrophage activation syndrome (MAS), severe inflammatory arthritis, chronic inflammation, fever, Kabuki syndrome, immune disorder, chronic inflammation/bone lesions, Crohn’s disease, capillary leak syndrome, refractory epilepsy, renal insufficiency, autoimmune ataxia	**59.9**	**53.2**	**76.9**	**55.3**	**41.4**	**48.3**	**75.2**
A2	COVID19, refractory epilepsy, febrile infection-related epilepsy syndrome (FIRES), systemic arthritis/MAS, fever, autoinflammatory disease, autoinflammatory syndrome	**69.5**	**41.4**	**46.4**	**34.4**	**71.0**	**70.2**	**57.3**
A3	Multisystem inflammatory syndrome in children (MIS-C) encephalitis, acute lymphocytic leukemia (ALL), post-hematopoietic stem cell transplant (HSCT) inflammation, Kawasaki disease, polyarthritis, lymphangioma, Castleman’s disease	**55.9**	***80.0***	**65.1**	**34.0**	**56.1**	**55.2**	***11.1***
A4	Multisystemic disease, paraneoplastic inflammatory state (mesothelioma), autoinflammatory disease, cryopyrin-associated periodic syndromes (CAPS), early onset inflammatory bowel disease (IBD), refractory epilepsy, fever, periodic fever/neutropenia, immunodeficiency workup	**46.6**	**50.8**	***0.4***	**34.4**	**29.0**	**38.4**	**32.3**

Diagnoses/reasons for testing are listed as they were provided on the test requisition forms submitted with the specimens. Median percentile rank values are highlighted by quintile: 0-20% (low ***blue italic text***); 20-40% (moderate low **blue text**); 40-60% (median **black text**); 60-80% (moderate high **red text**); 80-100% (high ***red italic text***).

**Table 4:** Adult clusters.

Patient cluster	Diagnosis/Reason for testing	Cytokine cluster (median percentile rank)								
		1		2	3	4	5	6	7
18-39 years									
B1	Adult onset Still’s disease (AOSD)/macrophage activation syndrome (MAS), hemophagocytic lymphohistiocytosis (HLH), HLH/MAS, autoinflammatory syndrome, autoimmune progesterone dermatitis (APD), recurrent fever, interferonopathy?, autoimmune lymphoproliferative syndrome (ALPS)	**57.2**		**59.8**	***18.4***	**38.2**	**55.2**	**74.7**	**43.9**
B2	None provided	**63.1**		**31.5**	**32.3**	**41.3**	**59.2**	**28.8**	**60.9**
B3	Chronic graft *vs* host disease (cGVHD), Muckle Wells syndrome?, familial Mediterranean fever (FMF), hidradenitis suppurativa	**46.2**		**49.4**	**73.1**	**50.4**	***81.3***	**40.8**	**55.6**
B4	Eosinophilia, granulomatous encephalitis, Behçet/autoinflammation, hidradenitis suppurativa, autoinflammatory syndrome, morphea, airway obstruction	**52.7**		**43.5**	**70.5**	**46.2**	**42.7**	**57.8**	**50.0**
B5	Refractory AOSD	**53.3**		**38.2**	**59.0**	**48.8**	**36.8**	**76.7**	**45.3**
40-59 years									
C1	Pyoderma gangrenosum, autoimmune myositis, chronic inflammation, eosinophilic abscesses, Crohn’s disease, autoinflammatory syndrome, AOSD	**53.1**		**40.2**	**71.9**	**57.8**	***82.1***	**67.2**	**37.4**
C2	Morphea, eosinophilia, inflammatory syndrome, ulcers, hidradenitis/Crohn’s	**63.9**		**53.7**	***82.9***	**49.0**	**34.9**	**56.0**	**21.6**
C3	Cryopyrin-associated periodic syndromes (CAPS), primary HLH/MAS, AOSD, TB HLH, adrenalitis	**51.3**		***82.1***	**31.3**	**46.0**	**43.7**	**34.3**	***19.6***
C4	Refractory autoimmune disease, polycythemia/thrombocytosis, crystal storing histiocytosis	**29.6**		**42.3**	**59.2**	**69.4**	**30.9**	**52.7**	**47.2**
C5	Systemic autoinflammation	**69.0**		**44.9**	**26.3**	**32.1**	**34.9**	***81.9***	**38.0**
C6	GVHD, lymphocyte variant hypereosinophilic syndrome (L-HES), Wells syndrome, severe inflammation of unknown origin, autoinflammatory syndrome, eosinophilic fasciitis, fever of unknown origin, giant cell arteritis, fibro-inflammatory masses, recurrent fever, chronic urticaria	**27.4**		**36.0**	***0.3***	**50.5**	**46.2**	**22.5**	**56.6**
60+ years									
D1	Sarcoidosis, episodic eosinophilia, Sweet syndrome	**55.2**		**76.4**	***86.0***	**73.7**	**46.6**	**44.5**	**55.1**
D2	AOSD, long COVID, hidradenitis suppurativa	**38.4**		**56.8**	**59.2**	**57.0**	**78.8**	**38.2**	**76.4**
D3	HES/Sjögrens, AOSD flare, HLH testing, chronic lymphocytic leukemia, morphea, FMF, bilateral hygroma, polyarthralgia, AOSD/FMF, Jak2+Cytosis, Jak2+ myeloproliferative syndrome, chemotherapy-induced fever, Castleman’s?	**39.2**		**53.9**	***0.3***	**41.3**	**51.5**	**29.8**	**45.1**

Diagnoses/reasons for testing are listed as they were provided on the test requisition forms submitted with the specimens. Median percentile rank values are highlighted by quintile: 0-20% (low ***blue italic text***); 20-40% (moderate low **blue text**); 40-60% (median **black text**); 60-80% (moderate high **red text**); 80-100% (high ***red italic text***).

Demographic data associated with each patient cluster are summarized in **Table 5**. There were no significant differences in the demographic data between the age group-specific clusters and their respective total cohorts, with the exception of the 40-59 years age group, in which cluster C1 had a significantly higher proportion of females (p= 0.0086), and cluster C5 had a significantly younger median age (p= 0.027) than the total age group cohort.

**Table 5:** Demographic characteristics of the patient clusters.

Cluster	n	Median age (range)	P value	Sex ratio (M:F)	P value
A1	26	7 (0.5 - 17)	>0.99	12:14	0.43
A2	21	10 (1 - 17)	0.49	11:10	1.0
A3	8	7 (0.5 - 17)	>0.99	5:3	0.74
A4	11	5 (0.5 - 15)	>0.99	8:3	0.37
Total 0-17	66	8.5 (0.5 - 17)		36: 30	
B1	23	31 (18 - 39)	>0.99	10:13	0.84
B2	9	27 (20 - 39)	0.49	4:5	1.0
B3	7	35 (23 - 39)	>0.99	4:3	0.46
B4	14	28 (18 - 38)	>0.99	5:9	0.79
B5	10	31.5 (24 - 39)	>0.99	3:7	0.54
Total 18-39	63	31 (18 - 39)		26:37	
C1	10	42 (40 - 55)	0.15	1:9*	0.0086
C2	11	50 (40 - 58)	>0.99	6:5	1.0
C3	7	52 (46 - 57)	>0.99	6:1	0.13
C4	12	50.5 (43 - 59)	>0.99	5:7	0.57
C5	7	43 (41 - 45)*	0.034	2:5	0.27
C6	33	50 (42 - 59)	>0.99	22:11	0.12
Total 40-59	80	48.5 (40 - 59)		42:38	
D1	9	71 (61 - 80)	>0.99	5:4	1.0
D2	9	65 (60 - 82)	>0.99	6:3	0.52
D3	27	66 (60 - 87)	>0.99	13:14	0.70
Total 60+	45	66 (60 - 87)		24:21	

Demographic data that are significantly different compared to the total age-specific cohort are indicated with asterisks (*).

**Figure 4:**
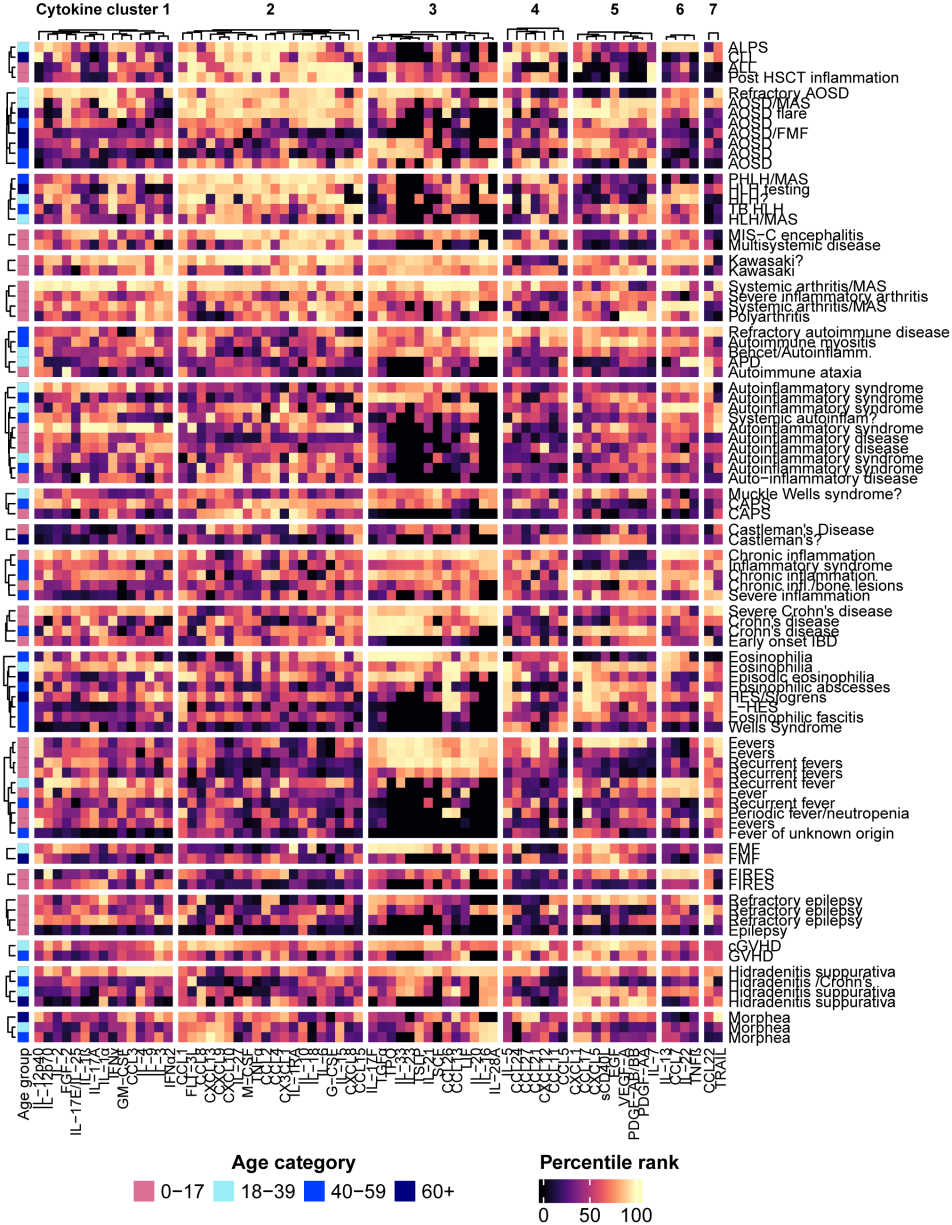
The patients were grouped according to the diagnoses or reasons for testing provided on the test requisitions, and hierarchical clustering of the patients within each group was performed. Each row represents an individual patient with the associated diagnosis or reason for testing listed on the right side of the heatmap.

Since patients with the same condition were often grouped into different patient clusters, patterns of disease-associated cytokine expression were visualized in groups of patients manually selected based on the diagnoses/reasons for testing provided on the test requisitions (**Figure 4**). Certain conditions appeared to be associated with consistent cytokine expression profiles based on a visual assessment of the heatmap: Lymphoproliferative syndromes (lymphocytic leukemia, autoimmune lymphoproliferative syndrome, HLH) tended to be associated with higher concentrations of the analytes in cytokine cluster 2 and moderate to low values in clusters 3 and 5; systemic inflammatory diseases in children (multisystem inflammatory syndrome in children (MIS-C), Kawasaki disease and juvenile arthritis) were associated with higher values in each of cytokine clusters 1, 2, 3, and 6; patients with Crohn’s disease (but not early-onset IDB) tended to be associated with high values in cytokine cluster 3; morphea was consistently associated with higher levels of certain chemokines in cytokine cluster 2, including CCL1, CCL8, CXCL13, and the IFN-induced chemokines CXCL9 and CXCL10. As observed in the patient clusters, other conditions were each associated with multiple cytokine expression profiles, such as AOSD, hidradenitis suppurativa, fevers, and eosinophilic diseases.

## 4 Discussion

A cohort of plasma specimens provided for clinical diagnostic testing by multiple specialists from Canada and the USA, representing individuals with a wide spectrum of confirmed or suspected inflammatory, autoimmune, neoplastic and neurological conditions, clusters of co-expressing cytokines were identified utilizing machine learning methods. Seven distinct cytokine clusters associated with different adult and pediatric disease groups suggesting there are common patterns of immune activation that are readily identifiable by cytokine multiplex array testing. Through the implementation of an evidence-based approach to cytokine testing, clinicians can utilize these biomarkers to improve diagnostic precision, thereby promoting a deeper comprehension of the pathogenic mechanisms and prognosis for these diseases, and ultimately facilitating the development of more effective treatments.

### 4.1 Cytokine clusters

Clusters 1 and 6 contain several cytokines linked to the development, activation or function CD4+ T cells, such as IL-2 [proliferation and clonal expansion of Th1 and Th2 cells (20)], IL-12p70 [differentiation and activation of Th1 cells (21)], IL-1 [differentiation of Th17 cells when present with IL-23 (22)], as well as several effector cytokines known to be released by T helper cell subtypes [Th1-IFNγ, IL-2, GM-CSF, TNFβ; Th2-IL-4, IL-13, IL-9; Th17 - IL-17A, IL-22; Th9-IL-9; Th22-IL-22 (5)]. Therefore, high levels of the analytes in these groups could indicate activation of adaptive immune responses. Many of the factors in cluster 2 are involved in innate immune responses and have been identified as mediators of cytokine release syndrome (CRS/cytokine storm), including IL-6, IL-10, IL-18, CXCL13, CXCL8, CXCL9, CXCL10, CCL2, CCL4, and TNFα (23). Analytes in cluster 3 represent cytokines involved in type 2 (IL-33, TSLP) and type 3 (IL-17F, IL-21, IL-23) immune responses, as well as factors that can drive or potentiate the expansion and differentiation of leukocytes and lymphocytes, such as stem cell factor (SCF) (24), thrombopoietin (TPO) (25), and IL-16 (26). There are also several analytes in cluster 3 that are prominently derived from or that act primarily upon epithelial cells, such as TSLP (27), IL-33 (28), TGFα (29), IL-28A (30), and IL-20 (31), which suggests an underlying functional basis for this grouping. Cluster 4 consists mainly of chemokines, of which CCL21, CCL27, and CXCL12 are known to be constitutively expressed homeostatic chemokines (32). Cluster 5, in which growth factors are prominently represented, likely represents a wound healing and/or platelet activation response, as these factors are generally stored in and released by platelets (33).

Interestingly, the cytokines known to contribute to adaptive T helper cell-mediated responses do not segregate based on immunotype. Factors associated with type 1, type 2 and type 3 immune responses are found in each of cluster 1 (type 1-IFNα2, IFNγ, IL-2, IL-12p70, CCL3 (34); type 2-IL-4, IL-9, IL-17E/IL-25; type 3-IL-1, IL-17A, IL-17F), cluster 3 (type 1-IL-28A; type 2 –CCL26, IL-33, TSLP; type 3-IL-21, IL-23, IL-17F), and cluster 6 (type 1-TNFβ; type 2-IL-13; type 3-IL-22). Since cytokines of one T cell immunotype often suppress the differentiation or function of other T cell immunotypes, these groupings may indicate common patterns of both immune activation and regulation. For example, the major Th17-released cytokine IL-17A clusters together with IL-2 and IFNα2, which both suppress Th17 differentiation and function (20, 35). These patterns of mixed T cell cytokine release could also reflect the heterogeneity and plasticity observed in T cells *in vivo*, since “hybrid” cells that co-express the signature cytokines of two different T cell subtypes (e.g., IL-4 with IFNγ, IFNγ with IL-17A, IL-4 with IL-17A, IL-13 expressed by both Th1 and Th17 cells) may be common (36–38).

### 4.2 Patient clusters

The pathological significance of these cytokine clusters in this cohort is limited due to the lack of detailed clinical information associated with the specimens, as well as low sample numbers associated with any one specific disease or condition. However, some potentially informative patterns were revealed with the patient clustering analyses. The largest category of known diagnoses or reasons for testing of the patients in this cohort consisted of conditions that are often associated with cytokine storm, such as adult-onset Still’s disease (AOSD) (39), hemophagocytic lymphohistiocytosis (HLH) (40), macrophage activation syndrome (MAS) (41), COVID-19 (42), multisystem inflammatory syndrome in children (MIS-C) (43), and hematopoietic stem cell transplantation (HSTC) (44), as well as Kawasaki disease, which has been shown to involve many of the same cytokines as those described in cytokine storm (45). The majority of the patients with these conditions had high values of cluster 2 analytes (many found in patient clusters A3, B2, and C3), which could indicate that this cytokine cluster includes the major mediators of cytokine storm. Higher values of the analytes in cluster 2 are also associated with lymphoproliferative syndromes (acute and chronic lymphocytic leukemia, autoimmune lymphoproliferative syndrome (ALPS)) and juvenile arthritis in this cohort.

The abundance of the analytes in cytokine cluster 3 may also provide diagnostic or prognostic value. High values in this cytokine cluster are observed in patient clusters, A1, A3, B3, B4, C1, C2, and D1, and tend to be associated with conditions such as arthritis, Kawasaki disease and Crohn’s disease (but not early-onset IBD), as well as subsets of the patients that were tested for AOSD, fever, autoimmune diseases, non-specified autoinflammatory disorders and eosinophilic conditions. These analytes were clustered in an analysis of cytokines released by peripheral blood mononuclear cells (PBMCs) isolated from COVID-19 patients (11). In that study high levels of these analytes were associated with greater disease severity, coagulopathy, and mortality, so it would be interesting to assess if similar outcomes apply to the other conditions with high values of the cluster 3 analytes.

Cytokine cluster 5, comprised of analytes that are released by activated platelets, may provide insight into the involvement of platelets in different conditions. High values in this cytokine cluster were observed in patient clusters A2, B3, C1, and D2, and associated with conditions that often involve thrombocytosis, such as AOSD (46), Kawasaki disease (47), juvenile arthritis (48), familial Mediterranean fever (FMF) (49), COVID-19 (50), and Crohn’s disease (51), whereas conditions that are generally associated with thrombocytopenia, such as HLH (52), lymphocytic leukemia (53), and hematopoietic stem cell transplantation (54) tended to have lower values. Further validation studies could help to clarify whether this cluster of cytokines has prognostic value for conditions in which abnormalities in platelet production and function are associated with disease severity.

There are some conditions that presented with different immune activation patterns (such as AOSD, fever of unknown origin, autoinflammatory disease) in this cohort. As this study only includes single samples from unique individuals, the temporal dynamics of cytokine expression related to immune activity and disease progression were not considered. The distinct cytokine profiles associated with individual diseases could be representative of different stages of disease progression, severity, exacerbation, etc. Furthermore, there was limited information provided of the treatments that were prescribed for these patients, which could have effects on the observed immune activation profiles. Explorations of the relationships between cytokine profiles and clinical presentations, disease stage/severity, treatments and treatment responses, and outcomes would therefore be an important future direction, particularly as they pertain to treatment options and whether different treatments may be more or less effective for inflammation characterized by different immune activation patterns. Other sources of the variability of the cytokine profiles observed for specific conditions could be due to the influence of patient demographic status on immune responses, and more detailed studies on these factors would also be of value. For example, in this cohort only children tested for fever, and only adults tested for autoinflammatory syndromes, had high values in cytokine cluster 3. It would be interesting to assess if these age-related differences are consistently observed in larger cohorts. Similarly, the expression of inflammatory and anti-inflammatory mediators has been found to change in post-menopausal women (55), so assessing the influence of menopausal status on these immune signatures in women may be an interesting and important factor requiring additional in-depth study. Other characteristics known to influence immune responses that could not be studied in this dataset due to a lack of patient-specific information, such as body mass index (BMI) (56), could also be a focus of future studies into the clinical significance of cytokine clusters. Additionally, although there is a range of diverse conditions represented in this cohort, there are likely other immune activation patterns that were not included in the present study cohort. Additional immune activation patterns may be revealed as further data are collected.

The main strength of this study is that the cohorts studied were representative of the samples received by a clinical diagnostic laboratory. We did not control for age, sex, or clinical presentation for this analysis, and as such the immune activation patterns that we identified are likely common in inflammatory and autoimmune conditions, and are likely be observed in future clinical test results. Hence, further exploration and validation of these results in separate highly characterized cohorts is required. The weakness of this study design is that the information that can be associated with the samples is limited to that provided on the test requisition forms, so we are unable to make strong inferences about the clinical relevance of the clusters with respect to diagnoses or clinical outcomes. Also, as the samples were collected remotely, there were possible variables in sample collection and transport (e.g., samples left thawed for varying periods of time before freezing) that we were unable to control but which reflects real world diagnostic serology. Further controlled studies on selected patient cohorts of specimens using strict standardized operating procedures would be useful to determine any potential effects of these potential variables.

In summary, we have identified novel clusters of co-expressing cytokines and common patterns of immune activation in a cohort of patient specimens provided for diagnostic cytokine testing. These clusters and immune signatures may provide clinicians with additional information regarding the inflammatory environment underlying the clinical presentation of their patients, and therefore could be a valuable resource in supporting diagnosis and identifying treatment options.

## Data availability statement

The raw data supporting the conclusions of this article will be made available by the authors, without undue reservation.

## Ethics statement

Ethical review and approval was not required for the study on human participants in accordance with the local legislation and institutional requirements. Written informed consent from the participants’ legal guardian/next of kin was not required to participate in this study in accordance with the national legislation and the institutional requirements.

## Author contributions

DP conceived of the study, conducted the literature review and wrote the manuscript drafts; PL and KB provided study design advice and biostatistics oversight; MC, KB, MJF, and MLF edited the manuscript, through to the final version. All authors read and approved the final submission.

## Glossary

**Table:**

*Diseases/conditions*	
ALL	acute lymphocytic leukemia
ALPS	autoimmune lymphoproliferative syndrome
AOSD	adult-onset Still’s disease
APD	autoimmune progesterone dermatitis
CAPS	cryopyrin-associated periodic syndrome
CLL	chronic lymphocytic leukemia
FIRES	febrile infection-related epilepsy syndrome
FMF	familial Mediterranean fever
GVHD	graft *vs*. host disease
cGVHD	chronic GVHD
HES	hypereosinophilic syndrome
L-HES	lymphocyte-variant HES
HLH	hemophagocytic lymphohistiocytosis
HSCT	hematopoietic stem cell transplantation
IBD	inflammatory bowel disease
MAS	macrophage activation syndrome
MIS-C	multisystem inflammatory syndrome in children
*Cytokines/growth factors*	
EGF	epidermal growth factor
FGF	fibroblast growth factor
FLT-3L	Fms-related tyrosine kinase 3 ligand
IFN	interferon
IL	interleukin
G-CSF	granulocyte colony stimulating factor
GM-CSF	granulocyte macrophage colony stimulating factor
LIF	leukemia inhibitory factor
M-CSF	macrophage colony stimulating factor
PDGF	platelet-derived growth factor
SCF	stem cell factor
TGF	transforming growth factor
TPO	thrombopoietin
TNF	tumor necrosis factor
TRAIL	TNF-related apoptosis-inducing ligand
TSLP	thymic stromal lymphopoietin
VEGF	vascular endothelial growth factor
